# Supplementary material for: Reporting of Perinatal Outcomes in Probiotic Randomized Controlled Trials. A Systematic Review and Meta-Analysis
Source: Nutrients. 2021 Jan 17;13(1):256. doi: 10.3390/nu13010256 (PMC7830438; doi:10.3390/nu13010256)
Supplement: Supplementary file 1 [file nutrients-13-00256-s001.zip › Supplementary 1.pdf]

Supplementary 1. Search strategy

PUBMED

1 - ("probiotic s"[All Fields] OR "probiotical"[All Fields] OR "probiotics"[MeSH Terms] OR "probiotics"[All Fields] OR "probiotic"[All Fields] OR ("probiotic s"[All Fields] OR "probiotical"[All Fields] OR "probiotics"[MeSH Terms] OR "probiotics"[All Fields] OR "probiotic"[All Fields])) AND ("pregnancy"[MeSH Terms] OR "pregnancy"[All Fields] OR "pregnancies"[All Fields] OR "pregnancy s"[All Fields]) (753 results)

2 - ("lactobacillus"[MeSH Terms] OR "lactobacillus"[All Fields]) AND ("pregnancy"[MeSH Terms] OR "pregnancy"[All Fields] OR "pregnancies"[All Fields] OR "pregnancy s"[All Fields]) (899 results)

3 - ("gestate"[All Fields] OR "gestated"[All Fields] OR "gestates"[All Fields] OR "gestating"[All Fields] OR "gestational"[All Fields] OR "gestations"[All Fields] OR "pregnancy"[MeSH Terms] OR "pregnancy"[All Fields] OR "gestation"[All Fields]) AND ("probiotic s"[All Fields] OR "probiotical"[All Fields] OR "probiotics"[MeSH Terms] OR "probiotics"[All Fields] OR "probiotic"[All Fields]) (955 results)

4 - ("gestate"[All Fields] OR "gestated"[All Fields] OR "gestates"[All Fields] OR "gestating"[All Fields] OR "gestational"[All Fields] OR "gestations"[All Fields] OR "pregnancy"[MeSH Terms] OR "pregnancy"[All Fields] OR "gestation"[All Fields]) AND ("lactobacillus"[MeSH Terms] OR "lactobacillus"[All Fields]) (1,001 results)

5 - ("gravidity"[MeSH Terms] OR "gravidity"[All Fields] OR "pregnant"[All Fields] OR "pregnants"[All Fields]) AND ("probiotic s"[All Fields] OR "probiotical"[All Fields] OR "probiotics"[MeSH Terms] OR "probiotics"[All Fields] OR "probiotic"[All Fields]) (283 results)

6 - ("randomized controlled trial"[Publication Type] OR "randomized controlled trials as topic"[MeSH Terms] OR "randomized clinical trial"[All Fields] OR "randomised clinical trial"[All Fields]) AND ("probiotic s"[All Fields] OR "probiotical"[All Fields] OR "probiotics"[MeSH Terms] OR "probiotics"[All Fields] OR "probiotic"[All Fields]) (3,119 results)

7 - "RCT"[All Fields] AND ("probiotic s"[All Fields] OR "probiotical"[All Fields] OR "probiotics"[MeSH Terms] OR "probiotics"[All Fields] OR "probiotic"[All Fields]) (152 results)

SCOPUS

1 - (TITLE-ABS-KEY ("Probiotics") AND TITLE-ABS-KEY ("pregnancy")) (1,234 results)

2 - (TITLE-ABS-KEY ("bifidobacterium") AND TITLE-ABS-KEY ("pregnancy")) (470 results)

3 - (TITLE-ABS-KEY ("lactobacillus") AND TITLE-ABS-KEY ("pregnancy")) (1,457 results)

4 - (TITLE-ABS-KEY ("probiotics") AND TITLE-ABS-KEY ("pregnant")) (467 results)

5 - (TITLE-ABS-KEY ("synbiotic") AND TITLE-ABS-KEY ("pregnancy")) (91 results)

COCHRANE LIBRARY (CENTRAL) (Trials)

1 - MeSH descriptor: [Probiotics] explode all trees (1973 results)

2 - MeSH descriptor: [Pregnancy] explode all trees (21,062 results)

3 - MeSH descriptor: [lactobacillus] explode all trees (1,579 results)

4 - MeSH descriptor: [bifidobacterium] explode all trees (713 results)

5 - MeSH descriptor: [Synbiotics] explode all trees (724 results)
