# Supplementary material for: Reporting of Perinatal Outcomes in Probiotic Randomized Controlled Trials. A Systematic Review and Meta-Analysis
Source: Nutrients. 2021 Jan 17;13(1):256. doi: 10.3390/nu13010256 (PMC7830438; doi:10.3390/nu13010256)
Supplement: Supplementary file 1 [file nutrients-13-00256-s001.zip › Supplementary 2.pdf]

Table S1. Risk of Bias Assessment.

Reference	Randomization process	Intended intervention	Missing outcome data	Outcome measurement	Selection of reported results	Overall bias
Abrahamsson et al. 2007 [29]	Low	Low	Low	Low	Some Concerns	Low
Ahmadi et al. 2016 [30]	Low	Low	Low	Low	Low	Low
Ali Pourmirzaiee et al. 2020 [31]	Some Concerns	Low	Low	Low	Low	Low
Allen et al. 2009 [32]	Low	Low	Some Concerns	Low	High	High
Asemi et al. 2011 [33]	Some Concerns	High	Low	Some Concerns	Some Concerns	High
Asgharian et al. 2019 [34]	Low	Low	Low	Low	Low	Low
Bababi et al. 2018 [35]	Low	Low	Low	Low	Low	Low
Badehnossh et al 2017 [36]	Low	Low	Low	Low	Low	Low
Boyle et al. 2010 [36]	Low	Low	Low	Low	Low	Low
Callawa7 et al. 2018 [38]	Low	Low	Low	Low	Low	Low
Chen et al. 2019 [14]	Some Concerns	High	Low	High	High	High
Dewanto et al. 2017 [39]	Some Concerns	Some Concerns	Some Concerns	Low	High	High
Dolatkhan et al. 2015 [40]	Low	Low	Low	Low	High	High
Dotterud et al. 2010 [41]	Some Concerns	Low	Some Concerns	Low	Low	Some Concerns
Fernández et al. 2015 [42]	Some Concerns	Some Concerns	Low	Some Concerns	Low	High
Gille et al. 2016 [43]	Low	Low	Low	Low	Low	Low
Halkjær et al. 2020 [44]	Low	Low	Low	Low	Low	Low
Ho et al. 2015 [45]	Some Concerns	Some Concerns	Low	Some Concerns	Low	High
Jafarnejad et al. 2016 [46]	Low	Low	Low	Some Concerns	High	High

Jamilian et al. 2016 [47]	Low	Low	Low	Some Concerns	Low	Some Concerns
Jamilian et al. 2018 [48]	Some Concerns	Low	Low	Some Concerns	High	High
Kalliomäki et al. 2001 [49]	Low	Low	Low	Low	Some Concerns	Some Concerns
Karamali et al. 2016 [50]	Low	Low	Low	Some Concerns	Low	Some Concerns
Karamali et al. 2017 [51]	Some Concerns	Low	Low	Some Concerns	Low	Some Concerns
Kijmanawat et al. 2018 [52]	Low	Low	Low	Low	Low	Low
Kim et al. 2009 [53]	Some Concerns	Low	Some Concerns	Low	Low	Some Concerns
Kopp et al. 2007 [54]	Some Concerns	Low	Low	Some Concerns	Low	Some Concerns
Kukkonen et al. 2006 [55]	Low	Low	Low	Low	Low	Low
Laitinen et al. 2008 [56]	Low	Low	Low	Low	Low	Low
Lindsay et al. 2014 [57]	Low	Low	Low	Low	Low	Low
Lindsay et al. 2015 [58]	Low	Low	Low	Low	Low	Low
Mantaring et al. 2018 [59]	Low	Some Concerns	Low	Some Concerns	Low	Some Concerns
Mastromarino et al. 2015 [61]	High	Low	High	Low	Low	High
McMillan et al. 2018 [63]	High	Low	High	Low	Low	High
Nabhani et al. 2018 [64]	Some Concerns	Low	Low	Some Concerns	Low	Some Concerns
Niers et al. 2009 [65]	Some Concerns	Low	Some Concerns	Low	Low	Some Concerns
Okense-Gafa et al. 2018 [66]	Low	Low	Low	Low	Low	Low
Olsen et al. 2017 [67]	High	High	Low	Some Concerns	High	High
Ou et al. 2012 [68]	Low	Low	Some Concerns	Some Concerns	Low	Some Concerns
Pellonperä et al. 2019 [69]	Low	Low	Low	Low	Low	Low
Sahhaf et al. 2019 [70]	Low	Low	Low	Some Concerns	Low	Some Concerns
Sharpe et al. 2019 [71]	Low	Low	Low	Some Concerns	Some Concerns	Some Concerns
Taghizadeh et al. 2013 [72]	Low	Low	Low	Low	Some Concerns	Some Concerns

Wickens et al. 2008 [73]	Low	Low	Low	Low	Low	Low
Wickens et al. 2017 [74]	Low	Low	Low	Low	Some Concerns	Some Concerns
Yang et al. 2020 [75]	Some Concerns	Low	Some Concerns	Some Concerns	Low	High
