# Supplementary material for: Reporting of Perinatal Outcomes in Probiotic Randomized Controlled Trials. A Systematic Review and Meta-Analysis
Source: Nutrients. 2021 Jan 17;13(1):256. doi: 10.3390/nu13010256 (PMC7830438; doi:10.3390/nu13010256)
Supplement: Supplementary file 1 [file nutrients-13-00256-s001.zip › Supplementary 3.pdf]

Table S2. Reporting of perinatal outcomes in individual studies

Author / Country / Year of acceptance for publication / Trial registration	Miscarriage or stillbirth	Weeks of gestation at delivery	Preterm birth	Birth weight	Large for gestational age	Macrosomia	Small for gestational age	Low birth weight	Cesarean section
Abrahamsson et al. / Sweden / 2007 / Not reported [29]	--	--	--	Probiotic = 3,658 Placebo = 3,603 (Statistic not specified)	--	--	--	--	Probiotic = 11 Placebo = 15
Ahmadi et al. / Iran / 2016 / IRCT201605085623N77 [30]	--	--	--	--	--	--	--	--	--
Ali Pourmirzaiee et al. / Iran / 2020 / IRCT201311004014882N7 [31]	Probiotic = 0 Placebo = 1 (Exclusion criterium)	Probiotic = 39.35 (0.64) Placebo = 39.48 (0.52) Mean (SD)	Probiotic = 3 Placebo = 5 (Exclusion criterium)	--	--	--	--	--	Probiotic = 41 Placebo = 41
Allen et al. / United Kingdom / 2009 / ISRCTN26287422 [32]	--	--	--	Probiotic = 3,490 (2,100-4,900) Placebo = 3,550 (2,000 – 5,200) Median (Range)	--	--	--	--	--
Asemi et al. / Iran / 2011 / Not reported [33]	--	--	--	--	--	--	--	--	--

Asgharian et al. / Iran / 2019 / IRCT201604013706N31 [34]	Probiotic = 1 Control = 1	--	Probiotic = 3 Control = 8	Probiotic = 3,270 (495) Control = 3,260 (435) Mean (SD)	Probiotic = 13 Control = 13	Probiotic = 3 Control = 3	--	--	Probiotic = 33 Control = 35
Bababi et al. / Iran / 2008 / IRCT20171010036697N1 [35]	--	--	--	--	--	--	--	--	--
Badehnoosh et al. / Iran / 2017 / IRCT201611115623N91 [36]	--	--	Probiotic = 2 Placebo = 1	Probiotic = 3,322 (444) Placebo = 3,438 (398) Mean (SD)	Probiotic = 9 Placebo = 5	Probiotic = 0 Placebo = 3	--	--	Probiotic = 6 Placebo = 14
Boyle et al. / Australia / 2008 / Cochrane Skin Group Trial No. 36 [37]	Probiotic = 0 Placebo = 1 (Exclusion criterium)	Probiotic = 39.6 (35.4 – 42.0) Placebo = 39.5 (36.0 – 42.3) Median (Range)	--	Probiotic = 3,560 (2,324 – 4,970) Placebo = 3,615 (2,105 – 5,020) Median (Range)	--	--	--	--	Probiotic = 14 Placebo = 12
Callaway et al. / Australia / 2018 / ACTRN12611001208998 [38]	Probiotic = 0 Placebo = 1	Probiotic = 39.32 (1.75) Placebo = 39.14 (1.88) Mean (SD)	Probiotic = 17 Placebo = 12	Probiotic = 3,524 (540) Placebo = 3,541 (514) Mean (SD)	Probiotic = 7 Placebo = 6	Probiotic = 31 Placebo = 30	Probiotic = 5 Placebo = 13	Probiotic = 7 Placebo = 6	Probiotic = 73 Placebo = 80
Chen et al. / China / 2019 / Not reported [14]	--	Probiotic = 39.6 (0.9) Placebo = 39.6 (1.1) Mean (SD)	--	Probiotic = 3,300 (470) Placebo = 3,300 (370) Mean (SD)	--	--	--	--	Probiotic = 4 Placebo = 1

Dewanto et al. / Indonesia / 2017 / Not reported [39]	--	--	--	--	--	--	--	--	Probiotic = 13<br>Placebo = 8<br>(Exclusion criterium)
Dolatkhan et al. / Iran / 2015 / IRCT201405181597N3 [40]	--	--	--	--	--	--	--	--	--
Dotterud et al. / Norway / 2010 / NCT00159523 [41]	--	--	Probiotic = 14<br>Placebo = 8	Probiotic = 3,671 (484)<br>Placebo = 3,595 (487)<br>Mean (SD)	--	--	--	--	--
Fernández et al. / Spain / 2015 / NCT01505361 [42]	--	--	--	--	--	--	--	--	--
Gille et al. / Germany / 2016 / ISRCTN40042090 [43]	Probiotic = 12<br>Placebo = 5	--	Probiotic = 6<br>Placebo = 8	--	--	--	--	--	--
Halkjær et al. / Denmark / 2020 /NCT02508844 [44]	--	Probiotic = 274 (19)<br>Placebo = 280 (11)<br>Mean (SD)	Probiotic = 4<br>Placebo = 0	Probiotic = 3,414 (676)<br>Placebo = 3,640 (454)<br>Mean (SD)	Probiotic = 1<br>Placebo = 4	Probiotic = 4<br>Placebo = 7	Probiotic = 1<br>Placebo = 0	--	Probiotic = 11<br>Placebo = 5
Ho et al. / Taiwan / 2015 / NCT01577108 [45]	--	Probiotic = 39 (0.9)<br>Placebo = 39 (1.1)<br>Mean (SD)	--	Probiotic = 3,123 (398)<br>Placebo = (358)<br>Mean (SD)	--	--	--	--	--
Jafarnejad et al. / Iran / 2016 / Not reported [46]	--	--	Probiotic = 2<br>Placebo = 1	--	--	--	--	--	--

			(Exclusion criterium) (<35 gw)						
Jamilian et al. / Iran / 2016 / IRCT201503035623N38 [47]	--	--	--	--	--	--	--	--	--
Jamilian et al. / Iran / 2018 / IRCT201706075623N119 [48]	--	Probiotic = 38.9 (2.5) Vitamin D and Probiotic = 39.3 (0.8) Placebo = 38.6 (1.1) Mean (SD)	Probiotic = 1 Vitamin D + Probiotic = 0 Placebo = 1	Probiotic = 3,171 (622) Vitamin D + Probiotic = 3,308 (604) Placebo = 3,176 (712) Mean (SD)	--	Probiotic = 1 Vitamin D + Probiotic = 2 Placebo = 5	--	--	Probiotic = 10 Vitamin D + Probiotic = 7 Placebo = 12
Kalliomaki et al. / Finland / 2001 / Not reported [49]	--	Probiotic = 39 (1.3) Placebo = 39 (1.4) Mean (SD)	--	Probiotic = 3,631 (483) Placebo = 3,612 (466) Mean (SD)	--	--	--	--	--
Karamali et al. / Iran / 2016 / IRCT201601035623N63 [50]	--	--	--	Probiotic = 3,253 Placebo = 3,386 (Mean)	--	Probiotic = 1 Placebo = 3 (Macrosomia not defined)	--	--	Probiotic = 7 Placebo = 11
Karamali et al. / Iran / 2017 / IRCT201704205623N108 [51]	--	Synbiotic = 39 (1.1) Placebo = 39.4 (1.5) Mean (SD)	Synbiotic = 1 Placebo = 0	Synbiotic = 3,373 (412) Placebo = 3,182 (460) Mean (SD)	--	Synbiotic = 0 Placebo = 3	--	--	Synbiotic = 5 Placebo = 12

Kijmanawat et al. / Thailand / 2018 / Thai Clinical Trials Registry Number 20170606002 [52]	--	--	--	Probiotic = 3,120 (411)<br>Placebo = 3,123 (370)<br>Mean (SD)	--	--	--	--	--
Kim et al. / Korea / 2009 / ISRCTN26134979 [53]	--	Probiotic = 39.7 (0.25)<br>Placebo = 39,5 (0.2)<br>Mean (Standard error)	--	Probiotic = 3,330 (70)<br>Placebo = 3,250 (60)<br>Mean (Standard error)	--	--	--	--	Probiotic = 5<br>Placebo = 11
Kopp et al. / Germany / 2007 / UKF000505 [54]	--	Probiotic = 39.8<br>Placebo = 39.7<br>Mean	--	Probiotic = 3,493<br>Placebo = 3,379<br>Mean	--	--	--	--	Probiotic = 9<br>Placebo = 9
Kukkonen et al. / Finland / 2006 / Not reported [55]	--	--	Probiotic = 27<br>Placebo = 18<br>(Exclusion criterium)	Probiotic = 3,595 (477)<br>Placebo = 3,591 (482)<br>Mean (SD)	--		--	--	Probiotic = 75<br>Placebo = 79
Laitinen et al. / Finland / 2008 / NCT00167700 [56,57]	Diet + probiotic = 3<br>Diet + placebo = 2<br>Control + Placebo = 0	Diet + Probiotics = 40 (36.9 – 42.8)<br>Diet + Placebo = 40 (30.5 – 42.4)<br>Control + Placebo = 40.0 (34.9 – 43.3)	Diet + probiotic = 2/80<br>Diet + placebo = 1/79<br>Placebo = 1/79	Diet + Probiotics = 3,467 (3,370 – 3,564)<br>Diet + Placebo = 3,579 (3,469 – 3,688)<br>Control + Placebo =	--	--	--	--	Diet + probiotic = 12/75<br>Diet + placebo = 12/77<br>Placebo = 11/76

		Median (Range)		3,611 (3,494 – 3,727) Mean (SD)					
Lindsay et al. / Ireland / 2014 / ISRCTN97241163(A) [58]	Probiotic = 0 Placebo = 0	Probiotic = 280 (10.2) Placebo = 282 (10.3) Mean (SD)	Probiotic = 3 Placebo 2	Probiotic = 3,700 (520) Placebo = 3,680 (510) Mean (SD)	Probiotic = 6 Placebo = 7	Probiotic = 15 Placebo = 16	--	--	Probiotic = 20 Placebo = 25
Lindsay et al. / Ireland /2015 / ISRCTN97241163(B) [59]	Probiotic = 0 Placebo 2	Probiotic = 278.34 (12.25) Placebo = 277.23 (11.20) Mean (SD)	Probiotic = 0 Placebo = 0	Probiotic = 3,570 (640) Placebo = 3,600 (570) Mean (SD)	Probiotic = 5 Placebo = 5	Probiotic = 19 Placebo = 17	Probiotic = 7/56 Placebo 7/58	--	Probiotic = 24 Placebo = 21
Mantaring et al. / Philippines / 2018 / NCT01073033 [60]	--	Supplement = 38.8 (1.5) Supplement + probiotic = 38.8 (2) Control = 38.8 (1.2) Mean (SD)	Supplement = 3/70 Supplement + probiotic = 1/70 Control = 1/68	Supplement = 2,930 (470) Supplement + probiotic = 2,900 (390) Control = 2,880 (440) Mean (SD)	--	--	--	--	Supplement = 15/70 Supplement + probiotic = 11/70 Control = 9/68
Mastromarino et al. / Italy / 2015 / NCT01367470 [61,62]	--	Probiotic = 39.4 (0.95) Placebo = 39 (1.1) Mean (SD)	-- (Exclusion criterium)	Probiotic = 3,350 (4,800) Placebo = 3,415 (501) Mean (SD)	--	--	--	--	Probiotic = 10 Placebo = 14
McMillan et al. / Rwanda / 2018 / NCT02150655 [63]	Probiotic = 0 Placebo = 1	--	Probiotic = 0 Placebo = 2	--	--	--	--	--	--
Nabhani et al. / Iran / 2018 /	--	--	Synbiotic= 1 Placebo = 1	--	--	--	--	--	--

IRCT201511183140N16 [64]			(<35 gw)						
Niers et al. / Netherlands/ 2009 / NCT00200954 [65]	--	Probiotic = 39.7 (39.2 – 40.2) Placebo = 39.7 (39.3 – 40.1) Mean (95%CI)	Probiotic = 1 Placebo = 2 (Exclusion criterium)	Probiotic = 3,558 (3,412 – 3,705) Placebo = 3,658 (3,520 – 3,796) Mean (95%CI)	--	--	--	--	Probiotic = 4 Placebo = 6
Okense-Gafa et al. / New Zealand / 2018 / ACTRN12615000400561 [66]	Probiotic = 2 Placebo = 2	Probiotic = 39.3 (1.7) Placebo = 38.9 (2.3) Mean (SD)	--	Probiotic = 3,685 (565) Placebo = 3,504 (672) Mean (SD)	--	--	Probiotic = 8 Placebo = 17	--	Probiotic = 40/112 Placebo = 35/114
Olsen et al. / Australia / 2017 / Not reported [67]	--	--	--	--	--	--	--	--	--
Ou et al. / Taiwan / 2012 / IDNCT00325273 [68]	--	Probiotic =39 (31 – 41) Placebo = 39 (35 – 41) Median (Range)	--	--	--	--	--	--	--
Pellonperä et. / Finland / 2019 / NCT01922791 [69]	Probiotic + Placebo = 1 Fish oil + Probiotic = 3 Fish oil + Placebo = 3	Probiotic + Placebo = 39.8 (1.4) Fish oil + Probiotic = 39.4 (2)	Probiotic + Placebo = 4 Fish oil + Probiotic = 11 Fish oil + Placebo = 4	Probiotic + Placebo = 3,620 (539) Fish oil + Probiotic = 3,530 (647)	Probiotic + Placebo = 13 Fish oil + Probiotic = 8 Fish oil + Placebo = 6	--	Probiotic + Placebo = 7 Fish oil + Probiotic = 8 Fish oil + Placebo = 7	--	Probiotic + Placebo = 14 Fish oil + Probiotic = 19 Fish oil + Placebo = 16

	Placebo + Placebo = 3	Fish oil + Placebo = 39.4 (2) Placebo + Placebo = 39.6 (1.4) Mean (SD)	Placebo + Placebo = 3	Fish oil + Placebo = 3.610 (516) Placebo + Placebo = 3,600 (503) Mean (SD)	Placebo + Placebo = 13		Placebo + Placebo = 9		Placebo + Placebo = 14
Sahhaf et al. / Iran / 2019 / IRCT20121224011862N2 [70]	--	Probiotic = 37.7 (1.9) Placebo = 38.1 (1.3) Mean (SD)	--	--	--	Probiotic = 2 Placebo = 8 (Macrosomia not defined)	--	--	--
Sharpe et al. / Canada / 2019 / NCT02528981 [71]	--	Probiotic = 40.0 (1.1) Placebo = 39.4 (1.5) Mean (SD)	--	--	--	--	--	--	Probiotic = 7 Placebo = 9
Taghizadeh et al. / Iran / 2013 / IRCT201212105623N3 [72]	--	--	--	--	--	--	--	--	--
Wickens et al. / New Zealand / 2008 / ACTRN12607000518460 [73]	--	--	--	Probiotic 1 = 3,480 (400) Probiotic 2 = 3,470 (500) Placebo = 3,480 (400)	--	--	--	--	Probiotic 1 = 46 Probiotic 2 = 57 Placebo = 50
Wickens et al. / New Zealand / 2017 / ACTRN12612000196842 [74]	--	Probiotic = 39.7 (38.7 – 40.7) Placebo = 39.6 (38.7 –	Probiotic = 16 Placebo = 8	Probiotic = 3,600 (3,500 – 3,700) Placebo = 3,500 (3,400	--	Probiotic = 46 Placebo = 32	--	--	Probiotic = 57 Placebo = 51

		40.4) Median (Interquartile range)		– 3,600) Mean (95%CI)					
Yang et al. / Canada / 2020 / NCT01697683 [75]	--	Probiotic = 39.1 (1.4) Placebo = 39.4 (0.9) Mean (SD)	Probiotic = 2 Placebo = 0	Probiotic = 3,340 (434) Placebo = 3,351 (464) Mean (SD)	--	--	--	--	Probiotic = 8 Placebo = 9

* Perinatal outcomes reported based on ICD-10 code chapter XV (pregnancy, childbirth and puerperium). Probiotic 10 / Placebo 13.
