# Supplementary material for: Reporting of Perinatal Outcomes in Probiotic Randomized Controlled Trials. A Systematic Review and Meta-Analysis
Source: Nutrients. 2021 Jan 17;13(1):256. doi: 10.3390/nu13010256 (PMC7830438; doi:10.3390/nu13010256)

Figure S1. Random effect meta-analysis of studies evaluating the administration of probiotics during early or mid-pregnancy and preterm birth

Study or Subgroup	Probiotic Events	Probiotic Total	Control Events	Control Total	Weight	Risk Ratio M-H, Random, 95% CI	Year
Laitinen, 2008	2	80	2	158	9.3%	1.98 [0.28, 13.76]	2008
Gille, 2016	6	160	8	160	22.2%	0.75 [0.27, 2.11]	2016
Wickens, 2017	16	205	8	201	27.8%	1.96 [0.86, 4.48]	2017
McMillan 2018	0	8	2	5	4.8%	0.13 [0.01, 2.32]	2018
Asgharian, 2019	3	64	8	64	17.2%	0.38 [0.10, 1.35]	2019
Pellonperä, 2019	4	96	3	92	14.3%	1.28 [0.29, 5.55]	2019
Yang 2020	2	41	0	43	4.4%	5.24 [0.26, 105.93]	2020
**Total (95% CI)**		**654**		**723**	**100.0%**	**1.03 [0.53, 1.99]**	
Total events	33		31				

Heterogeneity: Tau² = 0.23; Chi² = 8.64, df = 6 (P = 0.19); I² = 31%
Test for overall effect: Z = 0.09 (P = 0.93)

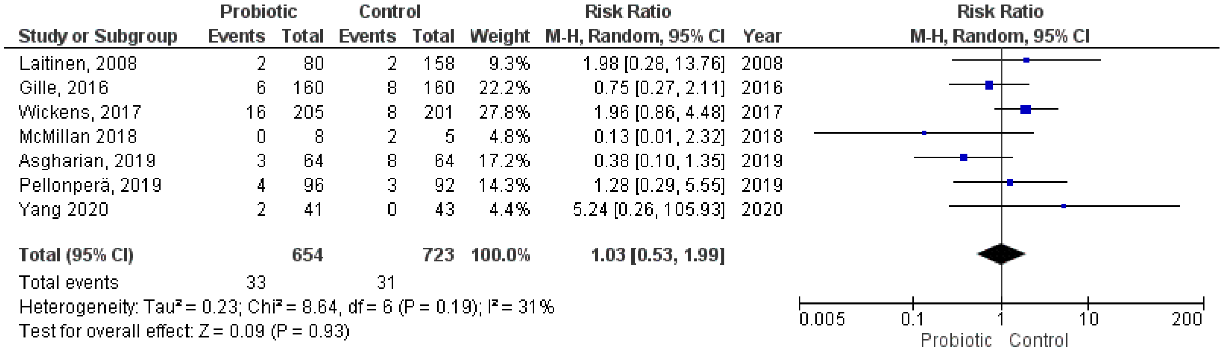

Figure S2. Random effect meta-analysis of studies evaluating the administration of *L.rhamnosus* GR-1 in combination with *L.reuteri* RC-14 and preterm birth

Study or Subgroup	Probiotic Events	Probiotic Total	Control Events	Control Total	Weight	Risk Ratio M-H, Random, 95% CI	Year
Gille, 2016	6	160	8	160	60.5%	0.75 [0.27, 2.11]	2016
McMillan 2018	0	8	2	5	20.5%	0.13 [0.01, 2.32]	2018
Yang 2020	2	41	0	43	19.0%	5.24 [0.26, 105.93]	2020
**Total (95% CI)**		**209**		**208**	**100.0%**	**0.76 [0.17, 3.35]**	
Total events	8		10				

Heterogeneity: Tau² = 0.67; Chi² = 3.02, df = 2 (P = 0.22); I² = 34%
Test for overall effect: Z = 0.36 (P = 0.72)

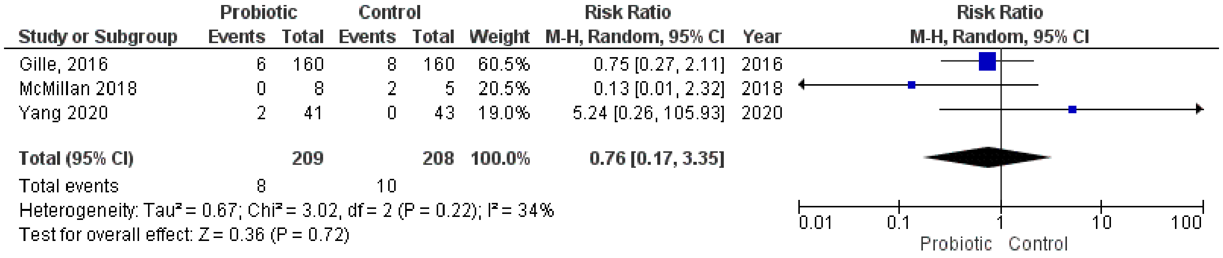

Figure S3. Random effect meta-analysis of studies evaluating the administration of probiotics and weeks of gestation reported as mean ± standard deviation.

Study or Subgroup	Probiotic Mean	Probiotic SD	Probiotic Total	Control Mean	Control SD	Control Total	Weight	Mean Difference IV, Random, 95% CI	Year
Kalliomaki 2001	39	1.3	77	39	1.4	82	13.8%	0.00 [-0.42, 0.42]	2001
Mastromarino 2016	39.4	0.95	33	39	1.1	33	11.7%	0.40 [-0.10, 0.90]	2016
Sahhaf 2019	37.7	1.9	42	38.1	1.3	42	7.7%	-0.40 [-1.10, 0.30]	2019
Sharpe 2019	40	1.1	57	39.4	1.5	56	11.9%	0.60 [0.11, 1.09]	2019
Pellonperä, 2019	39.8	1.4	96	39.4	1.4	92	14.3%	0.40 [-0.00, 0.80]	2019
Chen 2019	39.6	0.9	14	39.6	1.1	16	7.4%	0.00 [-0.72, 0.72]	2019
Ali Pourmirzaiee, 2020	39.4	0.6	74	39.5	0.5	71	21.7%	-0.10 [-0.28, 0.08]	2020
Yang 2020	39.1	1.4	41	39.4	0.9	43	11.4%	-0.30 [-0.81, 0.21]	2020
**Total (95% CI)**			**434**			**435**	**100.0%**	**0.09 [-0.14, 0.32]**	

Heterogeneity: Tau² = 0.06; Chi² = 15.85, df = 7 (P = 0.03); I² = 56%
Test for overall effect: Z = 0.75 (P = 0.45)

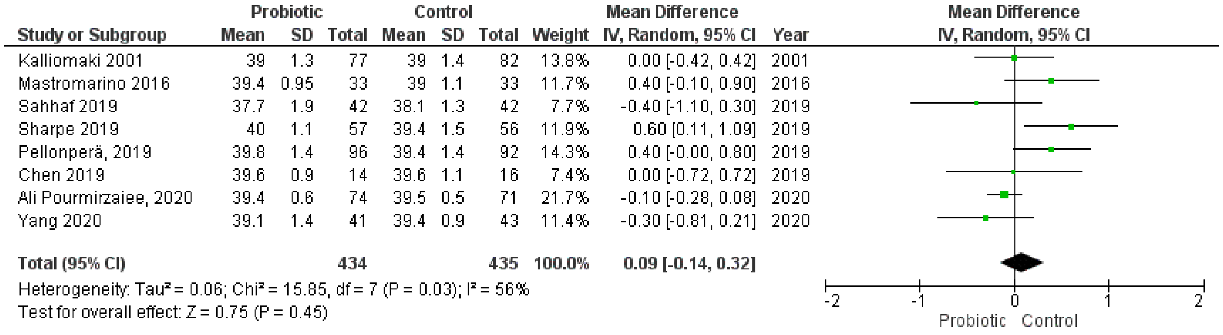

Figure S4. Random effect meta-analysis of studies evaluating the administration of *L.rhamnosus* GG and weeks of gestation

	Probiotic			Control				Mean Difference	
Study or Subgroup	Mean	SD	Total	Mean	SD	Total	Weight	IV, Random, 95% CI	Year
Kalliomaki 2001	39	1.3	77	39	1.4	82	32.7%	0.00 [-0.42, 0.42]	2001
Boyle 2008	39.1	1.3	125	39.3	1.2	125	36.1%	-0.20 [-0.51, 0.11]	2008
Ou 2012	37.5	2	95	38.5	1.2	96	31.2%	-1.00 [-1.47, -0.53]	2012
**Total (95% CI)**			**297**			**303**	**100.0%**	**-0.38 [-0.92, 0.15]**	

Heterogeneity: Tau² = 0.18; Chi² = 10.91, df = 2 (P = 0.004); I² = 82%
Test for overall effect: Z = 1.41 (P = 0.16)

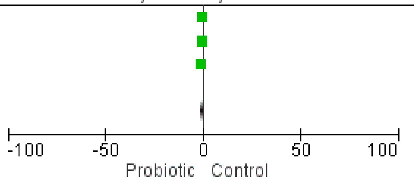

Figure S5. Random effect meta-analysis of studies evaluating the administration of *L.rhamnosus* GR-1 in combination with *L.reuteri* RC-14 and weeks of gestation

	Probiotic			Control				Mean Difference	
Study or Subgroup	Mean	SD	Total	Mean	SD	Total	Weight	IV, Random, 95% CI	Year
Sharpe 2019	40.01	1.14	57	39.36	1.45	56	50.3%	0.65 [0.17, 1.13]	2019
Yang 2020	39.1	1.4	41	39.4	0.9	43	49.7%	-0.30 [-0.81, 0.21]	2020
**Total (95% CI)**			**98**			**99**	**100.0%**	**0.18 [-0.75, 1.11]**	

Heterogeneity: Tau² = 0.39; Chi² = 7.11, df = 1 (P = 0.008); I² = 86%
Test for overall effect: Z = 0.38 (P = 0.71)

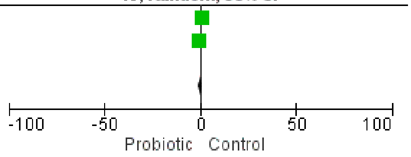

Figure S6. Meta-analysis of studies evaluating the administration of probiotic and birth weight reported as mean ± standard deviation.

Study or Subgroup	Probiotic Mean	Probiotic SD	Probiotic Total	Control Mean	Control SD	Control Total	Weight	Mean Difference IV, Random, 95% CI	Year
Kalliomaki 2001	3,631	483	77	3,612	466	82	6.9%	19.00 [-128.69, 166.69]	2001
Kukkonen, 2006	3,592	477	461	3,591	482	464	39.5%	1.00 [-60.80, 62.80]	2006
Wickens 2008	3,475	450	315	3,480	400	159	23.8%	-5.00 [-84.59, 74.59]	2008
Dotterud, 2010	3,671	484	138	3,595	487	140	11.6%	76.00 [-38.14, 190.14]	2010
Asgharian, 2019	3,270	495	64	3,260	435	64	5.8%	10.00 [-151.45, 171.45]	2019
Pellonperä, 2019	3,620	539	96	3,600	503	92	6.8%	20.00 [-128.96, 168.96]	2019
Chen 2019	3,300	470	14	3,300	370	16	1.6%	0.00 [-305.75, 305.75]	2019
Yang 2020	3,340	434	41	3,351	464	43	4.1%	-11.00 [-203.05, 181.05]	2020
**Total (95% CI)**			**1206**			**1060**	**100.0%**	**10.80 [-28.03, 49.62]**	

Heterogeneity: Tau² = 0.00; Chi² = 1.58, df = 7 (P = 0.98); I² = 0%
Test for overall effect: Z = 0.55 (P = 0.59)

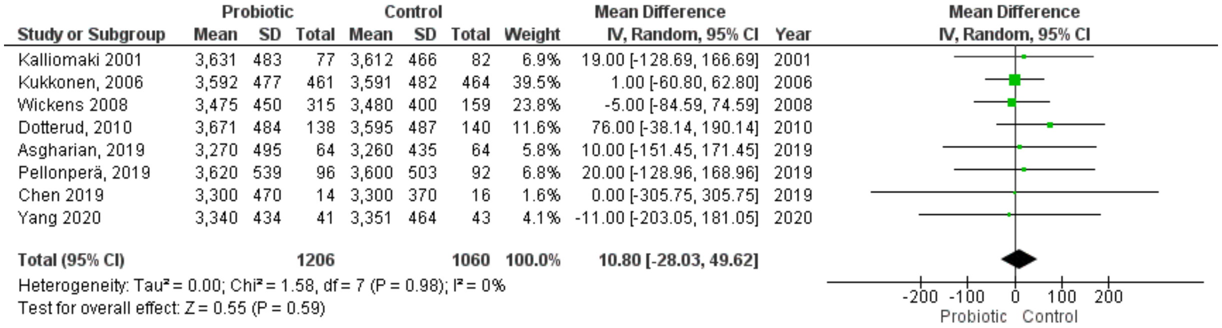

Figure S7. Random effect meta-analysis of studies evaluating the administration of probiotics during early or mid-pregnancy and birth weight

Study or Subgroup	Probiotic Mean	Probiotic SD	Probiotic Total	Control Mean	Control SD	Control Total	Weight	Mean Difference IV, Random, 95% CI	Year
Laitinen, 2008	3,467	450	85	3,595	524	171	25.4%	-128.00 [-251.77, -4.23]	2008
Wickens, 2017	3,600	726	205	3,500	721	202	20.9%	100.00 [-40.58, 240.58]	2017
Asgharian, 2019	3,270	495	64	3,260	435	64	16.8%	10.00 [-151.45, 171.45]	2019
Chen 2019	3,300	470	14	3,300	370	16	5.4%	0.00 [-305.75, 305.75]	2019
Pellonperä, 2019	3,620	539	96	3,600	503	92	19.1%	20.00 [-128.96, 168.96]	2019
Yang 2020	3,340	434	41	3,351	464	43	12.5%	-11.00 [-203.05, 181.05]	2020
**Total (95% CI)**			**505**			**588**	**100.0%**	**-7.43 [-80.27, 65.41]**	

Heterogeneity: Tau² = 1455.52; Chi² = 6.06, df = 5 (P = 0.30); I² = 18%
Test for overall effect: Z = 0.20 (P = 0.84)

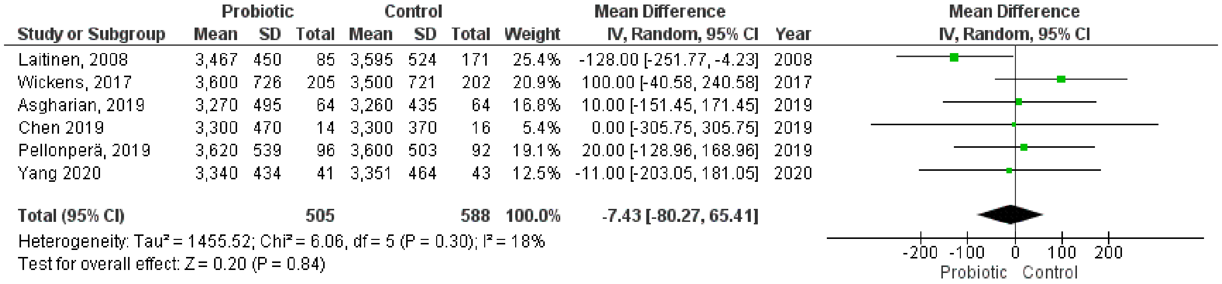

Figure S8. Random effect meta-analysis of studies evaluating the administration of *L.rhamnosus* GG and birth weight

Study or Subgroup	PROBIOTIC Mean	PROBIOTIC SD	PROBIOTIC Total	Control Mean	Control SD	Control Total	Weight	Mean Difference IV, Random, 95% CI	Year
Kalliomaki 2001	3,631	483	77	3,612	466	82	45.1%	19.00 [-128.69, 166.69]	2001
Boyle 2008	3,604	513	125	3,589	566	125	54.9%	15.00 [-118.91, 148.91]	2008
**Total (95% CI)**			**202**			**207**	**100.0%**	**16.80 [-82.40, 116.01]**	

Heterogeneity: Tau² = 0.00; Chi² = 0.00, df = 1 (P = 0.97); I² = 0%
Test for overall effect: Z = 0.33 (P = 0.74)

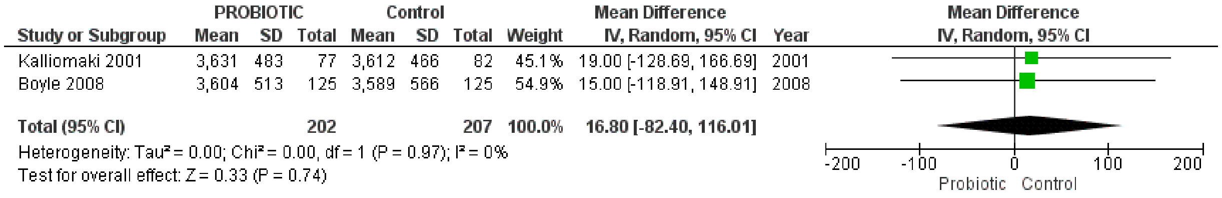

Figure S9. Random effect meta-analysis of studies evaluating the administration of *L.rhamnosus* GG and cesarean section

Study or Subgroup	Probiotic Events	Probiotic Total	Control Events	Control Total	Weight	Risk Ratio M-H, Random, 95% CI	Year
Kopp 2007	9	50	9	44	34.2%	0.88 [0.38, 2.02]	2007
Boyle 2008	12	33	14	32	65.8%	0.83 [0.46, 1.51]	2008
**Total (95% CI)**		**83**		**76**	**100.0%**	**0.85 [0.52, 1.38]**	
Total events	21		23				

Heterogeneity: Tau² = 0.00; Chi² = 0.01, df = 1 (P = 0.91); I² = 0%
Test for overall effect: Z = 0.67 (P = 0.50)

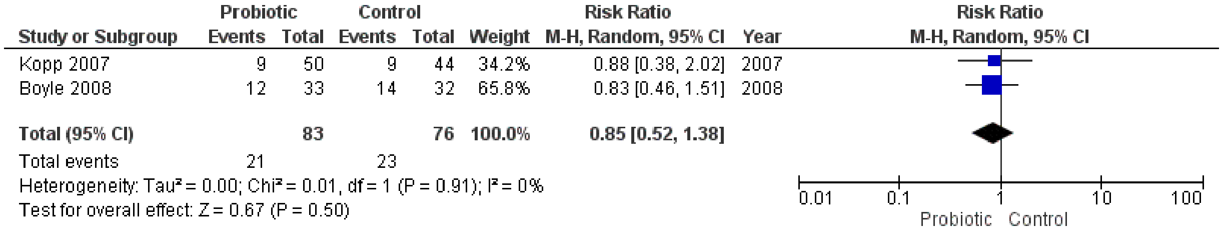

Figure S10. Random effect meta-analysis of studies evaluating the administration of *L.rhamnosus* GR-1 in combination with *L.reuteri* RC-14 and cesarean section

Study or Subgroup	Probiotic Events	Probiotic Total	Control Events	Control Total	Weight	Risk Ratio M-H, Random, 95% CI	Year
Sharpe 2019	7	57	9	56	46.3%	0.76 [0.31, 1.91]	2019
Yang 2020	8	41	9	43	53.7%	0.93 [0.40, 2.18]	2020
**Total (95% CI)**		**98**		**99**	**100.0%**	**0.85 [0.46, 1.59]**	
Total events	15		18				

Heterogeneity: Tau² = 0.00; Chi² = 0.10, df = 1 (P = 0.76); I² = 0%
Test for overall effect: Z = 0.51 (P = 0.61)

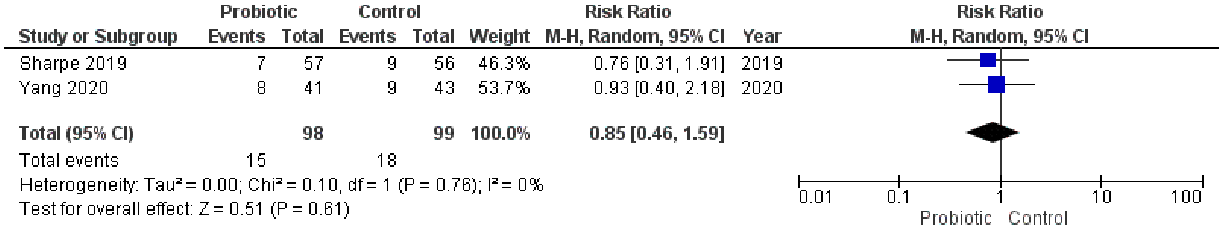

Supplement: Supplementary file 1 [file nutrients-13-00256-s001.zip › Supplementary 4.pdf]
